# Zinc Toxicity to Aminergic Neurotransmitters in Rat Brain

**DOI:** 10.4103/0971-6580.72670

**Published:** 2010

**Authors:** M. Vijaya Kumar, B. Nirmala Kumari, K. Yellamma

**Affiliations:** Department of Zoology, Sri Venkateswara University, Tirupati - 517502, Andhra Pradesh, India

**Keywords:** Aminergic system, behavioral changes, rat brain, zinc chloride

## Abstract

The present study was aimed to evaluate zinc toxicity to aminergic system in different areas of the brain of male albino rat, *Rattus norvegicus*. Zinc toxicity, evaluated as per Probit method was found to be 500 mg/kg body weight. For acute-dose studies, rats were given a single lethal dose of zinc chloride for one day only and for chronic-dose studies, the rats were administered with sub-lethal doses (1/10^th^ of lethal dose) of zinc chloride every day for 90 days continuously. Various constituents of the aminergic system viz. dopamine, norepinephrine, and epinephrine and the catabolizing enzyme, monoamine oxidase (MAO) were determined in different regions of rat brain such as olfactory lobe, hippocampus, cerebellum, and pons-medulla on selected time intervals/days under acute and chronic treatment with zinc. The results revealed that while the levels of all aminergic neurotransmitters were elevated differentially in the above mentioned areas of brain, MAO activity registered nonsignificant inhibition in all brain regions under zinc toxicity. All these changes in the aminergic system were subsequently manifested in the behavior of rat exhibiting the symptoms of mild tremors, reduced locomotor activity and emotions, restlessness followed by lacrymation, salivation, etc. From these observations, it was obvious that zinc treatment caused severe perturbations in the functions of the nervous system. Restoration of the aminergic system along with behavior to the near normal levels under chronic treatment indicates the onset of detoxification mechanisms or development of tolerance to zinc toxicity in the animal which was not probably so efficient under acute treatment.

## INTRODUCTION

Zinc is an important dietary constituent of all animals and is known to act as a neuroprotective agent and also as a modulator of the excitatory and inhibitory transmitters.[1] It is a part of at least 200 to 300 different metalloenzymes which are involved in a number of intracellular processes,[2] and acts as an essential requirement for proliferation of cells and for the successful growth of many internal organs and maturation of neurons[3] which are associated with general health of animals.[4] Liberation of intracellular zinc from cultured neurons[5] and the existence of zinc-containing pathways in telencephalon, hippocampus, brain stem, cerebellum, and amygdala[6] supported a physiological role for zinc in the mammalian Central Nervous System (CNS). Recent findings have demonstrated that peroxynitrite-induced neuronal apoptosis is mediated by intracellular release of zinc.[7] Zinc translocation accelerates infraction after mild transient focal ischemia,[8] brain injury,[9] and ageing of brain.[10] Identification of specific transporter genes for zinc provides strong evidences that zinc has a vital role in mammalian system.[11]

Zinc which is also a trace element is relatively nontoxic to birds and mammals, and a wide margin of safety is observed between normal intake and those likely to produce deleterious effects. However, experimental evidences proved that intake of higher concentration of zinc has a negative effect causing retarded growth, anemia, anorexia, heavy mortality,[12] and a number of neurological diseases such as Alzheimer’s disease, Picks disease, schizophrenia, epilepsy, etc.[13] Because zinc finds a wide range of commercial uses viz., paper industry, treating textiles, soldering, etc., prolonged exposure of human beings to zinc compounds poses occupational hazards. In view of the above observations in the present study, an attempt has been made to evaluate the toxic effects of zinc on the aminergic system in the brain of rat subjected to chronic and acute treatment, and manifestation of these changes in the behavior of rat.

## MATERIALS AND METHODS

Male albino rats, *Rattus norvegicus*, weighing 140 ± 10 g, obtained from Sri Venkateswara Enterprises, Bangalore, were selected as experimental animals, and zinc chloride (98%) from Loba Chemicals, Bombay, India was selected as the toxicant. The rats were fed with food pellets (Lipton Indian Ltd., Bangalore) and drinking water *ad libitum*. The animals were maintained in the laboratory conditions according to the instructions given by Behringer[14] 15 days before experimentation. Toxicity evaluation was done by Probit method of Finney.[15] Dopamine, norepinephrine, and epinephrine were assayed as per Kari *et al*.,[16] and monoamine oxidase (MAO) as per Green and Haughton.[17]

Biochemical estimations were done under acute and chronic exposures. For acute exposures, the animals were sacrificed at 3, 12, and 24 hours intervals after treatment with a single lethal dose of zinc chloride, and for chronic exposures, the animals were sacrificed on the 10^th^, 20^th^, 30^th^, 60^th^, and 90^th^ after treatment with sub-lethal doses of zinc chloride every day up to 90 days. After cervical dislocation, the brain was isolated quickly and placed in ice. Different areas of brain such as olfactory lobe, hippocampus, cerebellum, and pons-medulla were isolated by following standard anatomical marks[18] and were immediately homogenized in suitable media for biochemical analysis. The results obtained were analyzed statistically by following standard methods.

Observations on the overt behavior of rats treated with both acute and chronic doses of zinc chloride were made throughout the period of experimentation to coincide with the time intervals of aminergic system against the controls and used for correlation of the changes in aminergic systems and behavior of rat.

## RESULTS

### Dopamine, norepinephrine, and epinephrine

**Figure 1:**
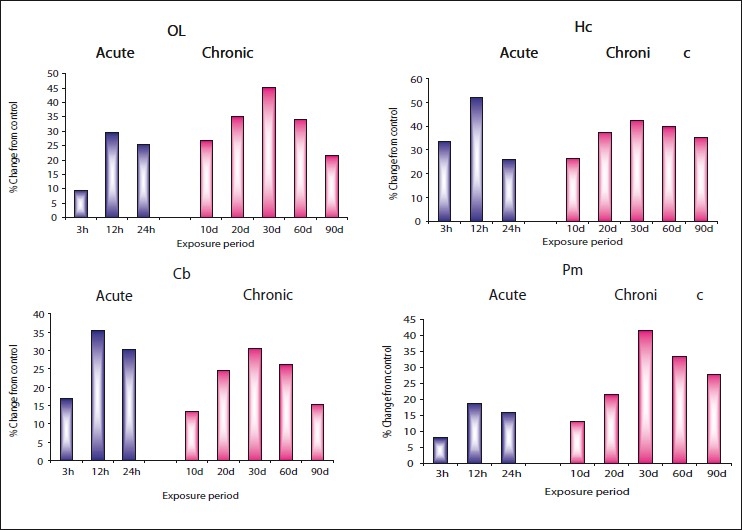
Per cent change from control in the *in vivo* content of dopamine (DA) in olfactory lobe (OL), hippocampus (HC), cerebellum (Cb), and pons-medulla (Pm) of rat brain following treatment with acute and chronic doses of zinc chloride

**Figure 2:**
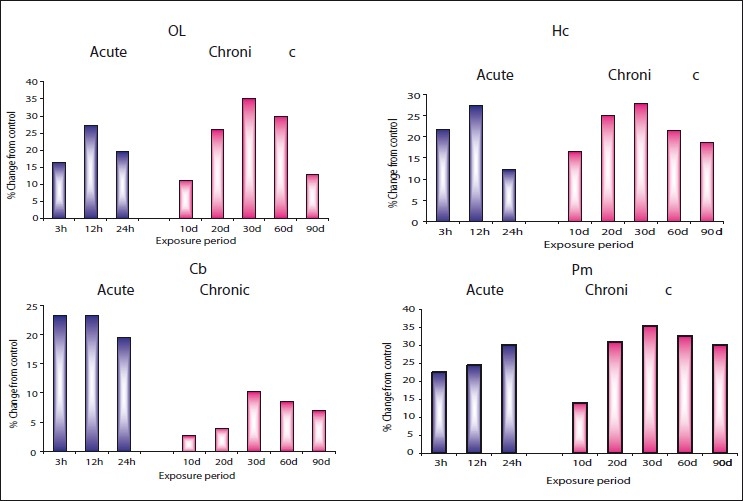
Per cent change from control in the *in vivo* content of norepinephrine (NEP) in olfactory lobe (OL), hippocampus (HC), cerebellum (Cb), and pons-medulla (Pm) of rat brain following treatment with acute and chronic doses of zinc chloride

**Figure 3:**
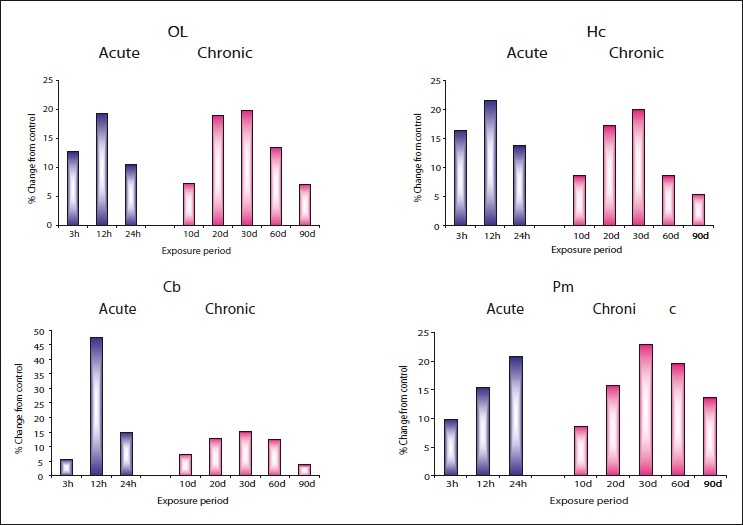
Per cent change from control in the *in vivo* content of epinephrine (EP) in olfactory lobe (OL), hippocampus (HC), cerebellum (Cb), and pons-medulla (Pm) of rat brain following treatment with acute and chronic doses of zinc chloride

The results [Figures 1 – 3 and Tables 1 – 3] clearly indicate that zinc chloride has significantly altered the levels of dopamine, norepinephrine, and epinephrine in all areas of rat brain such as olfactory lobe, hippocampus, cerebellum, and pons-medulla under both acute and chronic exposures. Dopamine and norepinephrine registered a similar trend of elevation in all brain areas of rat under acute and chronic exposures against the control. Maximum accumulation of norepinephrine was noticed in pons-medulla (30.13%) followed by hippocampus (27.30%), olfactory lobe (26.38%), and cerebellum (23.30%) at 24 hours during acute dose. Under chronic treatment also, all the brain areas showed significant elevation in norepinephrine on 30^th^ day, in the order of pons-medulla>olfactory lobe>hippocampus>cerebellum. Even though dopamine levels under acute treatment registered maximum elevation at different times of exposure, under chronic exposure, maximum accumulation in all the brain areas was evident on 30^th^ day and then showed recovery through 60 and 90 days.

**Table 1:** Changes in dopamine content (μg/g wet wt) in OL, HC, Cb, and Pm of male albino rats exposed to acute and chronic doses of Zncl_2_

	Acute						Chronic									
	C	3h	C	12h	C	24h	C	10d	C	20d	C	30d	C	60d	C	90d
OL	0.263 ± 0.003	0.238** ± 0.006	0.267 ± 0.007	0.215** ± 0.003	0.225 ± 0.011	0.190** ± 0.004	0.258 ± 0.008	0.189** ± 0.004	0.260 ± 0.006	0.169** ± 0.006	0.262 ± 0.003	0.144** ± 0.009	0.264 ± 0.203	0.174** ± 0.006	0.266 ± 0.002	0.209** ± 0.008
		(9.50)		(29.47)		(25.49)		(26.74)		(35.00)		(45.03)		(34.09)		(21.42)
HC	0.557 ± 0.008	0.370** ± 0.007	0.560 ± 0.006	0.268** ± 0.009	0.562 ± 0.004	0.415** ± 0.007	0.584 ± 0.008	0.430** ± 0.007	0.566 ± 0.003	0.354** ± 0.007	0.570 ± 0.008	0.328** ± 0.007	0.571 ± 0.008	0.344** ± 0.006	0.576 ± 0.006	0.372** ± 0.005
		(33.57)		(52.14)		(26.15)		(26.36)		(37.45)		(42.45)		(39.75)		(35.41)
Cb	0.202 ± 0.017	0.168** ± 0.007	0.206 ± 0.010	0.133** ± 0.007	0.204 ± 0.013	0.142** ± 0.003	0.208 ± 0.006	0.180** ± 0.007	0.212 ± 0.007	0.160** ± 0.007	0.216 ± 0.008	0.150** ± 0.006	0.214 ± 0.008	0.158** ± 0.004	0.209 ± 0.006	0.177** ± 0.006
		(16.83)		(35.43)		(30.39)		(13.46)		(24.52)		(30.55)		(26.16)		(15.31)
Pm	0.313 ± 0.005	0.288** ± 0.017	0.320 ± 0.005	0.260** ± 0.007	0.316 ± 0.003	0.266** ± 0.007	0.322 ± 0.002	0.280** ± 0.007	0.318 ± 0.002	0.250** ± 0.007	0.312 ± 0.006	0.183** ± 0.007	0.315 ± 0.003	0.210** ± 0.033	0.317 ± 0.003	0.229** ± 0.009
		(7.98)		(18.75)		(15.82)		(13.04)		(21.38)		(41.34)		(33.33)		(27.76)

Values are mean ± SD of six observations each from tissues pooled from six animals. Values in parentheses indicate per cent changes from control.

*** Values are significant at *P*<0.001.

** indicate significance at *P*<0.01.

* indicate significance at *P*<0.005.

# Not significant. SD - standard deviation; OL - olfactory lobe; HC - hippocampus; Cb- cerebellum; Pm - pons-medulla

**Table 2:** Changes in norepinephrine content (μg/g wet wt) in OL, HC, Cb and Pm of male albino rats exposed to acute and chronic doses of Zncl_2_

	Acute						Chronic									
	C	3h	C	12h	C	24h	C	10d	C	20d	C	30d	C	60d	C	90d
OL	1.137 ± 0.006	0.950** ± 0.008	1.140 ± 0.004	0.829** ± 0.005	1.160 ± 0.007	0.934** ± 0.007	1.170 ± 0.007	1.040** ± 0.005	1.150 ± 0.005	0.852** ± 0.005	1.186 ± 0.016	0.770** ± 0.006	1.179 ± 0.005	0.826** ± 0.040	1.145 ± 0.005	0.997** ± 0.049
		(16.44)		(27.28)		(19.48)		(11.11)		(25.91)		(35.07)		(29.94)		(12.92)
HC	1.132 ± 0.004	0.886** ± 0.016	1.128 ± 0.007	0.820** ± 0.015	1.136 ± 0.006	0.997** ± 0.018	1.148 ± 0.009	0.959** ± 0.008	1.134 ± 0.009	0.849** ± 0.008	1.149 ± 0.007	0.830** ± 0.018	1.144 ± 0.008	0.897** ± 0.013	1.131 ± 0.004	0.920** ± 0.013
		(21.73)		(27.30)		(12.23)		(16.46)		(25.13)		(27.76)		(21.59)		(18.65)
Cb	0.690 ± 0.011	0.530** ± 0.012	0.678 ± 0.014	0.520** ± 0.009	0.676 ± 0.015	0.544** ± 0.005	0.674 ± 0.014	0.656# ± 0.	0.664 ± 0.007	0.638** ± 0.008	0.675 ± 0.015	0.606** ± 0.016	0.670 ± 0.015	0.613** ± 0.010	0.677 ± 0.013	0.630** ± 0.007
		(23.18)		(23.30)		(19.52)		(2.67)		(3.91)		(10.22)		(8.50)		(6.94)
Pm	2.620 ± 0.005	2.030** ± 0.006	2.628 ± 0.007	1.984** ± 0.007	2.648 ± 0.005	1.850** ± 0.010	2.575 ± 0.013	2.216** ± 0.009	2.637 ± 0.005	1.820** ± 0.009	2.590 ± 0.029	1.670** ± 0.008	2.618 ± 0.008	1.765** ± 0.006	2.622 ± 0.005	1.831** ± 0.008
		(22.51)		(24.50)		(30.13)		(13.94)		(30.98)		(35.52)		(32.58)		(30.16)

Values are mean ± SD of six observations each from tissues pooled from six animals; Values in parentheses indicate per cent changes from control;

*** Values are significant at *P*<0.001;

** indicate significance at *P*<0.01;

* indicate significance at *P*<0.005;

# Not significant. SD - standard deviation; OL - olfactory lobe; HC - hippocampus; Cb- cerebellum; Pm - pons-medulla;

**Table 3:** Changes in epinephrine content (μg/g wet wt) in OL, HC, Cb, and Pm of male albino rats exposed to acute and chronic doses of Zncl_2_

	Acute						Chronic									
	C	3h	C	12h	C	24h	C	10d	C	20d	C	30d	C	60d	C	90d
OL	0.306 ± 0.005	0.267** ± 0.010	0.346 ± 0.009	0.413** ± 0.011	0.362 ± 0.020	0.400** ± 0.013	0.382 ± 0.020	0.410** ± 0.009	0.348 ± 0.007	0.414** ± 0.012	0.338 ± 0.006	0.405** ± 0.010	0.363 ± 0.210	0.412** ± 0.011	0.377 ± 0.210	0.404# ± 0.012
		(12.74)		(19.36)		(10.49)		(7.32)		(18.96)		(19.82)		(13.49)		(7.16)
HC	0.524 ± 0.013	0.610** ± 0.013	0.507 ± 0.010	0.616** ± 0.015	0.532 ± 0.016	0.605** ± 0.011	0.574 ± 0.017	0.623** ± 0.006	0.536 ± 0.009	0.628** ± 0.005	0.517 ± 0.012	0.620** ± 0.010	0.560 ± 0.009	0.608** ± 0.011	0.575 ± 0.013	0.606** ± 0.010
		(16.41)		(21.49)		(13.72)		(8.53)		(17.16)		(19.92)		(8.57)		(5.39)
Cb	0.229 ± 0.005	0.242** ± 0.007	0.162 ± 0.019	0.239** ± 0.010	0.215 ± 0.012	0.247** ± 0.010	0.235 ± 0.007	0.252# ± 0.023	0.227 ± 0.009	0.256** ± 0.021	0.218 ± 0.009	0.251** ± 0.025	0.226 ± 0.009	0.254** ± 0.022	0.241 ± 0.002	0.250# ± 0.026
		(5.67)		(47.53)		(14.88)		(7.23)		(12.77)		(15.13)		(12.38)		(3.73)
Pm	0.700 ± 0.028	0.769** ± 0.016	0.771 ± 0.011	0.652** ± 0.019	0.630 ± 0.008	0.761** ± 0.007	0.717 ± 0.011	0.779** ± 0.032	0.679 ± 0.018	0.786** ± 0.018	0.609 ± 0.006	0.749** ± 0.006	0.634 ± 0.008	0.758** ± 0.010	0.660 ± 0.025	0.750** ± 0.008
		(9.85)		(15.43)		(20.79)		(8.64)		(15.75)		(22.98)		(19.55)		(13.63)

Values are mean ± SD of six observations each from tissues pooled from six animals; Values in parentheses indicate per cent changes from control;

*** Values are significant at *P*<0.001;

** indicate significance at *P*<0.01;

* indicate significance at *P*<0.005;

# Not significant; SD - standard deviation; OL - olfactory lobe; HC - hippocampus; Cb - cerebellum; Pm - pons-medulla

In control rat brain, the distribution of epinephrine in different areas was in the order of pons-medulla (0.700), hippocampus (0.524), olfactory lobe (0.306), and cerebellum (0.229). In rats treated with acute doses of zinc chloride, though significant elevation in epinephrine in different areas of brain was observed from 3 hours onwards, maximum elevation of 47.53% in cerebellum at 12 hours; 32.67% in olfactory lobe at 3 hours; 21.49% in hippocampus at 12 hours; 20.79% in pons-medulla at 24 hours was recorded, suggesting area specific effect of zinc in rat brain. However, the onset of recovery in all brain areas was noticed at 24 hours.

Similarly, elevation in epinephrine was also noticed in all brain areas of rat treated with chronic doses of zinc chloride from 10^th^ day onwards. These changes were more pronounced after 20 days reaching peak levels on 30^th^ day in pons-medulla (22.98%), followed by hippocampus (19.92%), olfactory lobe (19.82%), and cerebellum (15.83%). However, these changes showed recovery tendency after 30 days.

### Monoamine oxidase

**Figure 4:**
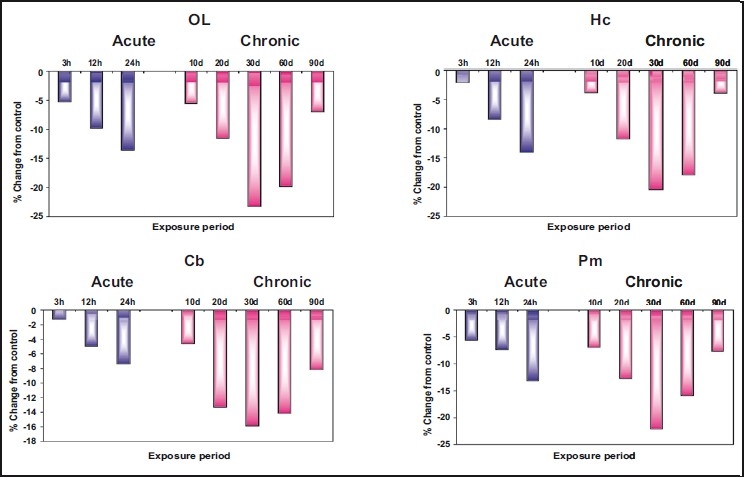
Per cent change from control in the *in vivo* content of monoamine oxidase (MAO) in olfactory lobe (OL), hippocampus (HC), cerebellum (Cb), and pons-medulla (Pm) of rat brain following treatment with acute and chronic doses of zinc chloride

Figure 4 and Table 4 show that while all the aminergic neurotransmitters recorded enhanced levels in various regions of brain under both acute and chronic treatments, MAO activity was inhibited by zinc. The changes in MAO were more pronounced in rats exposed to chronic doses than acute dose. Under chronic exposure, maximum inhibition in MAO was recorded on 30^th^ day in olfactory lobe (23.27%), followed by pons-medulla (22.10%), hippocampus (20.44%), and least in cerebellum (15.89%), whereas in acute dose maximum inhibition was at 24 hours in hippocampus (14.04%) and least in cerebellum (7.42%). However, recovery was observed from 24 hours and after 30 days in acute and chronic exposures respectively.

**Table 4:** Changes in monoamine oxidase activity (μmoles of p-hydroxy phenyl acetaldehyde formed/g wt/h) in OL, HC, Cb, and Pm of male albino rats exposed to acute and chronic doses of Zncl_2_

	Acute						Chronic									
	C	3h	C	12h	C	24h	C	10d	C	20d	C	30d	C	60d	C	90d
OL	3.420 ± 0.006	3.239** ± 0.006	3.450 ± 0.007	2.981** ± 0.006	3.435 ± 0.006	3.099** ± 0.012	3.460 ± 0.006	3.268** ± 0.006	3.480 ± 0.007	3.078** ± 0.006	3.459 ± 0.005	2.654** ± 0.005	3.442 ± 0.007	2.760** ± 0.007	3.424 ± 0.006	3.185# ± 0.012
		(-5.29)		(-13.59)		(-9.78)		(-5.54)		(-11.55)		(-23.27)		(-19.81)		(-6.98)
HC	3.584 ± 0.007	3.507** ± 0.006	3.560 ± 0.006	3.060# ± 0.006	3.594 ± 0.010	3.292** ± 0.007	3.559 ± 0.006	3.424** ± 0.007	3.572 ± 0.007	3.152** ± 0.007	3.556 ± 0.006	2.829** ± 0.007	3.558 ± 0.004	2.920** ± 0.007	3.588 ± 0.006	3.450** ± 0.007
		(-2.14)		(-14.04)		(-8.40)		(-3.79)		(-11.75)		(-20.44)		(-17.93)		(-3.84)
Cb	2.451 ± 0.007	2.419** ± 0.007	2.450 ± 0.005	2.268** ± 0.039	2.440 ± 0.007	2.319** ± 0.007	2.463 ± 0.005	2.349** ± 0.006	2.446 ± 0.006	2.120** ± 0.006	2.448 ± 0.005	2.059** ± 0.006	2.464 ± 0.007	2.115** ± 0.007	2.457 ± 0.007	2.256** ± 0.007
		(-1.30)		(-7.42)		(-4.95)		(-4.62)		(-13.32)		(-15.89)		(-14.16)		(-8.18)
Pm	3.755 ± 0.006	3.543** ± 0.008	3.751 ± 0.005	3.258** ± 0.006	3.765 ± 0.006	3.489** ± 0.007	3.780 ± 0.007	3.519** ± 0.006	3.757 ± 0.006	3.279** ± 0.007	3.746 ± 0.006	2.918** ± 0.008	3.756 ± 0.006	3.159** ± 0.007	3.758 ± 0.005	3.470** ± 0.003
		(-5.64)		(-13.14)		(-7.33)		(-6.90)		(-12.72)		(-22.10)		(-15.89)		(-7.66)

Values are mean ± SD of six observations each from tissues pooled from six animals; Values in parentheses indicate per cent changes from control;

*** Values are significant at *P*<0.001;

** indicate significance at *P*<0.01;

* indicate significance at *P*<0.005;

# Not significant; SD - standard deviation; OL - olfactory lobe; HC - hippocampus; Cb - cerebellum; Pm - pons-medulla

### Behavioral changes

The behavioral changes exhibited by the rat exposed to acute and chronic doses of zinc recorded at selected time intervals/days included adipsia (lack of drinking), aphagia (lack of eating), hypokinesia (reduced locomotor activity), mild tremors, emotions, restlessness followed by lacrymation, salivation, etc.

## DISCUSSION

Our observation in the present study emphasize that zinc chloride has induced significant and varied levels of elevation in dopamine, norepinephrine, and epinephrine and inhibited MAO in various regions of rat brain under both acute and chronic exposures to zinc chloride, substantiating that zinc might be affecting various steps in the metabolic pathway of the synthesis of these neurotransmitters via end-product inhibition which is maximal when neuronal activity and transmitters release are low, there by leading to high catecholamine concentration in tyrosine hydroxylase (TH) accessible pool.

The synthesis of aminergic neurotransmitters is regulated by a bewildering variety of processes, many of which operate via the rate-limiting enzyme TH. Some of the factors that regulate the synthesis of the neurotransmitters operate very rapidly, thereby allowing cells to respond to short-term needs. It should also be noted that studies on the control of these neurotransmitters synthesis have used a number of different model system, including adrenal medullary chromaffin cells, pheochromocytoma cells, sympathetic noradrenergic neurons, noradrenergic neurons of the locus coeruleus, and nigrostriatal dopaminergic neurons.[19] Earlier reports demonstrating that zinc is a part of at least 200 to 300 different enzymes which are involved in a number of intracellular processes[2] further substantiate that zinc might be modulating the key enzyme TH involved in the metabolism of these aminergic neurotransmitters. The mammalian CNS contains an abundance of the transition metal zinc which is highly localized in the neuronal parenchyma. Zinc is actively taken up and stored in synaptic vesicles in nerve terminals and stimulation of nerve fiber tracts that contain large amounts of zinc can induce its release, and thus modulates transient outward current gating in hippocampal neurons.[20] Known interaction of zinc with the major excitatory and inhibitory amino-acid neurotransmitter receptors in the CNS further supports this notion.[21] The movement and role of actively functioning zinc, i.e., vesicular zinc, in the amygdala revealed that zinc is localized in the limbic system, which may correspond to the regions with high densities of zinc-containing neuron terminals, suggesting that vesicular zinc is essential to the function of the amygdala, e.g., olfactory function.[6] Accumulation and release of zinc from synaptic regions,[22] liberation of intracellular zinc from cultured neurons,[5] the existence of zinc-containing pathways in telencephalon, hippocampus, brain stem, and cerebellum supported a physiological role for zinc in the CNS. Identification of specific transporter genes for zinc lends further support to the important role assigned for zinc in mammalian system.[11]

The inhibition in MAO indicates that zinc is blocking its release in this enzyme, leading to accumulation of the catecholamines at the nerve terminals. MAO is not only present in noradrenergic and dopaminergic cells, but also plays a central role in the metabolism of aminergic neurotransmitters. Cloning studies have recently identified the specific rat[23] and human vesicular transporter protein for noradrenergic, dopaminergic, and serotonergic cell groups in the brain, indicating that this one protein is probably responsible for accumulating all three amines in synaptic vesicles.[23]

Variable levels of elevation in these aminergic neurotransmitters in different brain regions were due to heterogeneous nature of the brain tissue and different roles assigned to different neurotransmitters such as norepinephrine and serotonin-motor hyperactivity.[24] Dopamine-complex stereotypy[25] or due to the disturbances in the cholinergic system.[26] However, the effects of AChE inhibitors on MAO levels in rat brain are confusing.[27] In the present study, the mechanisms other than MAO-mediated changes in catecholamine levels such as release mechanisms, conversions mediated by other monoamine enzymes such as COMT, etc. may be in operation during long-term exposure of rats to zinc chloride.[28]

The areas of rat brain exhibiting changes in cholinergic system are shown to exhibit the greatest changes in noncholinergic system,[27] thus indicating their possible interdependence. Thus, it is conceived that the adaptive changes underlying tolerance to anticholinesterase agents involve alterations in other neurotransmitter system as well in balance with the cholinergic system. The more recent observations of Takeda *et al*.[29] on hippocampal mossy fibers of rat demonstrated that the releases of zinc and glutamate and calcium signaling are interlinked during excitation cells.

The behavioral changes such as adipsia, aphagia, hypokinesia, etc. observed in rats under zinc toxicity revealed that zinc might have caused lesions in the important regions of brain like substantial nigra, hypothalamus, etc. These motor deficits and motivational changes are closely associated with some of the symptoms characteristic to Parkinson’s disease. Our observations in the present study give clear indications that continuous exposure to zinc compounds for prolonged durations might increase the risk of Parkinson’s disease among the working community in the industries. The noradrenergic neurons, in the pons-medulla are organized into three main clusters: the locus coeruleus complex (LC), the lateral tegmental system, and a dorsal medullary group. The LC, which is the most prominent noradrenergic nucleus, projects rostrally to virtually all regions of the telencephalon and diencephalons, dorsally to the cerebellum, and caudally to the spinal cord modulating a variety of important behavioral and physiological processes. The earlier reports demonstrating the existence of zinc-containing pathways in telencephalon, hippocampus, brain stem, and cerebellum not only support a physiological role for zinc in the CNS, but also give conclusive evidence that zinc exerts toxic effects in rats on prolonged exposure and disturbs the functions of the nervous system.

Furthermore, recovery tendency noticed in all aminergic neurotransmitters and MAO in all brain regions of rat and its behavior upon exposure to both acute and chronic doses of zinc chloride indicate the operation of the detoxification mechanisms and development of behavioral tolerance in rat. It further reveals that the signs and symptoms of zinc toxicity were not of cholinergic origin.

All trace elements exert toxic effects if ingested at sufficiently high levels for long durations. Zinc, which is also a trace element, is relatively nontoxic to birds and mammals and a wide margin of safety is observed between normal intake and those likely to produce deleterious effects. Suh *et al*.[30] reported that neurons exposed to zinc exhibit activation of poly (ADP-ribose) polymerase-1 (PARP-1), an enzyme that normally participates in DNA repair but promotes cell death when extensively activated. Besides this, intake of higher concentration of zinc produces a number of negative effects such as retarded growth, anemia, anorexia, and heavy mortality,[12] and a number of neurological diseases such as Alzheimer’s disease, Picks disease, schizophrenia, epilepsy, etc.[13]

All our observations in the present study provide conclusive evidences that the aspect of zinc toxicity to human beings need special attention from the environmentalist point of view to suggest proper precautionary measures to be implemented in places such as paper industry, treating textiles, soldering, etc., because zinc finds a wide range of commercial uses, and prolonged exposure of human beings to zinc compounds poses higher risk of occupational hazards.
